# Military Surgery in the French Army

**Published:** 1869-10

**Authors:** E. Andrews

**Affiliations:** Prof. of Principles and Practice of Surgery, Chicago Medical College


ARTICLE XXXVI.

### MILITARY SURGERY IN THE FRENCH ARMY.

By E. ANDREWS, M.D., Prof. of Principles and Practice of Surgery, Chicago Medical College.

The French Surgeons are much exercised at present over the enormous mortality of their military surgery, as compared with that of the English and Americans.

Last year, the *Gazette Hebdomadaire* published articles setting forth the frightful figures connected with the French surgery in the Crimean War. This year, Mons. le Dr. J. C. Chenu has published a work, in two large quarto volumes, with a folio atlas, on the surgical results of the last French campaign in Italy, at the time Napoleon III. expelled the Austrians from Lombardy. Notwithstanding that Northern Italy has a fine climate, lies close to the borders of France, and abounds in everything necessary for wounded men, the same frightful excess of mortality in French surgery is displayed, which was before seen in the Crimea. The following table illustrates the differences between the different armies:—

| OPERATIONS. | U. S. Army, War of Secession. | English Army, Crimean War. | FRENCH ARMY. Crimean War. | FRENCH ARMY. Italian War. |
|---|---|---|---|---|
| Disarticulation of Shoulder,..... | 39.2 | 33.3 | 61.7 | 52.7 |
| Amputation of Arm,............... | 21.2 | 24.5 | 55.5 | 55.8 |
| Amputation of Forearm, ........ | 16.5 | 5.0 | 45.2 | 42.8 |
| Disarticulation at Hip-joint, ... | 85.7 | 100.0 | 100.0 | 57.1 |
| Amputation of Thigh,............ | 64.4 | 64.0 | 91.8 | 76.4 |
| Amputation at Knee, ............ | 55.1 | 57.1 | 91.3 | 75.0 |
| Amputation of Leg, .............. | 26.0 | 35.6 | 71.9 | 66.5 |
| | 40.2 | 33.9 | 72.8 | 63.9 |

PER CENT. OF MORTALITY.

From this, it appears that the mortality after French military amputation has been about 60 per cent. greater than in the American army, and nearly one hundred per cent. greater than in the British. The *Gazette Hebdomadaire* takes up the controversy, and attributes this disastrous result to two main causes:—1st. The organization of the army which makes the surgeons dependent on the *Intendant* (a sort of Quartermaster) for supplies; in consequence of which the wounded were often short of good rations, and, 2d. The reckless transfer of patients from one hospital to another. It does not seem to me that the writer in the *Hebdomadaire* makes his points well. In the U. S. Armies, the Medical Department was absolutely dependant on the Quartermaster and Commissary for supplies, and partly so for transportation. All branches of the service are in the same condition in that respect, and, in the nature of the case, must be. In the turmoil of war, rations will get stopped or damaged occasionally; and yet we did not find that short or even damaged rations were half as injurious to the wounded as some other things to be presently mentioned. A similar criticism may be made respecting the assertion, that the transfer from hospital to hospital was necessarily a chief cause of mortality, and a reason why their men suffered more than ours.

No doubt, transportation injures some patients, but when done in open, airy, and not crowded conveyances, it does much less harm than we formerly supposed. On Sherman's march to the sea, the wounded were all carried through to Savanna in ambulances, shaking and jolting over bad roads, and yet the amputations recovered magnificently. No set of wounded men ever did better, evidently because they had the freshest of pure air. In active military operations, the hospitals near the front must often be abandoned and the patients transferred; besides, they become overcrowded, and require relief for that reason, otherwise, half the patients will die of typhus, erysipelas, hospital gangrene, and pyæmia. I believe that we had in our army more such transfers than the French, and yet we suffered less mortality, by 60 per cent. It is not difficult for an observant military surgeon to conjecture the true cause of the French ill success. There is but one thing which can produce so many deaths after amputations as M. Chenu describes, and that is overcrowding, or what amounts to the same thing, foul air and bad ventilation. Overcrowding, and consequent foul air, means putrefaction, erysipelas, hospital gangrene, pyæmia, and death. When surgeons lose 55 per cent. of their amputations of the arm, and seventy per cent. of their amputations below the knee, we know very well what that means. It is of no use to accuse the short rations, mouldy "hard tack," or rough transportation; such men have assuredly been overcrowded or under-ventilated. They have breathed the effluvium of each other's wounds, until their whole systems were permeated with the germs of putrefaction, and were ready to succumb to every operation, however slight. American surgeons tried that out on the large scale early in the war, but, fortunately, they had sense enough to learn from experience, and not to perpetuate their early blunders.

I have no doubt that the following is a true account of the matter. Frenchmen have very little comprehension of the amount of fresh air which wounded men require. Even Velpeau, that old giant of French surgery, was a wretched sinner against science in this respect. I well recollect walking the rounds of his hospital with him, and noticing that his wards literally stank with foul air. I was not at all surprised to notice in his hospital reports, that in the winter season (when windows are shut, and fresh air almost excluded) he had a regular annual epidemic of malignant erysipelas. When the men, field-officers, and surgeons of an army are alike destitute of any idea of the danger of ill-ventilation, death will reap a harvest out of their ranks. The plains of Lombardy are full of village and buildings of every description, erected for anything else but ventilation. I presume the wounded were crowded into these buildings, in the first place, as the nearest solid shelter. There they got their first poisoning. Then they were sent in ill-ventilated, crowded cars, by rail, to Genoa, and absorbed their second course of putrefacient germs. At Genoa, they were placed into, close, sea-going vessels, which are the most deadly and unventilable machines ever contrived for the destruction of wounded men, and taken a voyage to Marseilles, and thus drank their third course of putridity. If any of them did not die by this time, it was because they were proof against all ordinary causes of destruction. I presume that this, or something like it, was the true surgical history of the Italian campaign of Napoleon III. If the French surgeons, in their next war, will see to it that every wounded man, from the hour of battle to the day of his recovery or decease, breathes no air but that which is as fresh and pure as that in the sky, they will find that their statistics of amputations will compare favorably with those of any nation.

